# Activin A Signaling Regulates IL13Rα2 Expression to Promote Breast Cancer Metastasis

**DOI:** 10.3389/fonc.2019.00032

**Published:** 2019-02-05

**Authors:** Maria Kalli, Fotios Mpekris, Chen K. Wong, Myrofora Panagi, Sait Ozturk, Sam Thiagalingam, Triantafyllos Stylianopoulos, Panagiotis Papageorgis

**Affiliations:** ^1^Department of Life Sciences, European University Cyprus, Nicosia, Cyprus; ^2^Cancer Biophysics Laboratory, Department of Mechanical and Manufacturing Engineering, University of Cyprus, Nicosia, Cyprus; ^3^Genetics & Genomics and Pathology & Laboratory Medicine, Biomedical Genetics Section, Molecular Medicine Program and Cancer Center, Department of Medicine, Boston University School of Medicine, Boston, MA, United States

**Keywords:** Activin A, breast cancer, migration, metastasis, IL13Rα2

## Abstract

Metastatic dissemination of cancer cells to distal organs is the major cause of death for patients suffering from the aggressive basal-like breast cancer (BLBC) subtype. Recently, we have shown that interleukin 13 receptor alpha 2 (IL13Rα2) is a critical gene that is overexpressed in a subset of BLBC primary tumors associated with poor distant metastasis-free survival (DMFS) and can promote extravasation and metastasis of breast cancer cells to the lungs. However, the upstream signaling mechanisms that promote aberrant IL13Rα2 expression during tumor progression remain unknown. Driven by our previously published gene expression microarray data derived from a well-characterized cell line model for BLBC progression, we show that both Inhibin βA (INHBA) and IL13Rα2 genes exhibit similarly higher expression levels in metastatic compared to non-metastatic cells and that overexpression of both genes predicts worse metastasis-free survival of patients with high grade tumors. Activin A, a member of the TGFβ superfamily comprising two INHBA subunits, has been shown to play context-depended roles in cancer progression. Here, we demonstrate that INHBA depletion downregulates IL13Rα2 expression in metastatic breast cancer cells, whereas treatment with Activin A in non-metastatic cells increases its expression levels. We also find that Activin A predominantly induces Smad2 phosphorylation and to a lesser extent activates Smad3 and Akt. Interestingly, we also show that Activin A-mediated upregulation of IL13Rα2 is Smad2-dependent since knocking down Smad2 or using the ALK4/ALK5 inhibitors EW-7197 and SB-505124 abolishes this effect. Most importantly, our data indicate that knocking down INHBA levels in breast cancer cells delays primary tumor growth, suppresses migration *in vitro* and inhibits the formation of lung metastases *in vivo*. Conclusively, our findings presented here suggest that the development of therapeutic interventions employing small molecule inhibitors against Activin receptors or neutralizing antibodies targeting Activin A ligand, could serve as alternative approaches against breast tumors overexpressing INHBA and/or IL13Rα2.

## Introduction

Despite significant diagnostic and therapeutic advances, breast cancer remains the second leading cause of cancer-associated deaths in women worldwide (1). Basal-like breast cancer (BLBC) is considered one of the most aggressive subtypes that largely overlaps with triple-negative breast tumors. While metastatic dissemination to secondary organs accounts for the vast majority of patient deaths, the underlying molecular mechanisms have just recently begun to emerge (2, 3). The discovery of genes implicated in particular steps of the metastatic cascade, such as intravasation, extravasation, or metastatic colonization, has generated hopes for developing effective therapeutic interventions. Interleukin 13 receptor alpha 2 gene (*IL13R*α*2*) encodes for a decoy receptor which has high affinity for binding and internalization of IL13 ligand but is unable to transduce downstream signaling through the classical Signal Transducer and Activator of Transcription (STAT) signaling pathway (4). Therefore, increased IL13Rα2 levels have been shown to antagonize IL13-mediated responses via the canonical IL13Rα1-IL4Rα-STAT signaling axis (5, 6). Importantly, studies have also indicated that IL13Rα2 overexpression facilitates the progression and metastasis of various cancer types, including glioblastoma, head and neck, pancreatic, colorectal, and ovarian cancers (7–13). Recently, we and others have demonstrated that IL13Rα2 is also overexpressed in a subset of BLBC cell lines and primary tumors and could promote migration, extravasation and metastasis of breast cancer cells to the lungs (14, 15). However, the upstream signaling events that control and promote IL13Rα2 overexpression in BLBC remain unknown.

Activin A belongs to the transforming growth factor-beta (TGFβ) superfamily of cytokines which exert a plethora of biological functions, including developmental differentiation, sex determination, control of cellular proliferation, migration and immune responses (16–19). Structurally, it is a homodimer comprised of two Inhibin βA subunits, encoded by the *INHBA* gene, and becomes biologically active upon proteolytic cleavage of a pro-Activin A precursor molecule (20). Activin A initiates signaling by binding to a type II receptor (ActRII) followed by heterodimerization with a type I receptor (ActRI/ALK4 or ActRI/ALK2) (21–23). Activated ALK4 or ALK2 receptors recruit and phosphorylate Smad2 and/or Smad3 which form complexes with Smad4, translocate to the nucleus and regulate gene expression along with other transcriptional co-factors (24). Similar to other members of the TGFβ superfamily, such as TGFβ1, Activin A has been shown to play dual roles in cancer progression depending on the genetic and cellular context as well as tumor stage, exerting early tumor suppressive and late pro-metastatic effects (25, 26). Initial studies using the estrogen receptor positive (ER^+^) breast cancer cell line T47D demonstrated that Activin A could promote Smad-dependent cell cycle arrest (27), whereas more recent evidence suggested that Activin A overexpression could promote epithelial to mesenchymal (EMT) transition, invasion and metastasis of breast cancer (28). However, the molecular mechanisms and downstream target genes that mediate these events have not yet been elucidated.

Based on our previously published gene expression microarray data using a well-characterized human cell line model system for BLBC progression (14, 29), we show here that both INHBA and IL13Rα2 exhibit similarly higher expression levels in metastatic compared to non-metastatic cells and that overexpression of both genes predicts worse metastasis-free survival of patients with high grade tumors. Our data also demonstrate that Activin A signaling induces Smad-depended IL13Rα2 expression and that knocking down INHBA levels delays primary tumor growth and suppresses formation of lung metastases *in vivo*.

## Materials and Methods

### Cell Culture

MCF10A (MI), MCF10ATk1.cl2 (MII), MCF10CA1h (MIII), and MCF10CA1a (MIV) breast cancer cell lines were obtained by Karmanos Cancer Institute (Detroit, MI, USA) and were cultured in Dulbecco's Modified Eagle's Medium Nutrient Mixture-12 (DMEM/F12) medium supplemented with 5% Horse Serum (HS), 1% antibiotics, 10 mg/ml Insulin, 20 ng/ml Epidermal Growth Factor (EGF), 100 ng/ml Cholera Toxin (CT), and 500 ng/ml Hydrocortisone (HC). All cells were incubated at 37°C and 5% CO_2_ in a humidified incubator.

### Cell Treatments

To examine the effects of Activin A on gene expression, 5 × 10^5^ MII cells were seeded in 6-well plates in complete DMEM/F12 medium. After overnight incubation, cells were serum starved in 0.2% HS-containing DMEM/F12 medium overnight and then treated with 10 ng/ml human recombinant Activin A (R&D) for 6 or 16 h. Control cells were treated with equal volume of the Activin A solvent (4 mM HCl supplemented with 0.1% Bovine Serum Albumin-BSA). Similarly, to examine the signaling pathways regulated by Activin A, 5 × 10^5^ MII cells were seeded in 6-well plates in complete DMEM/F12 medium and then serum starved in 0.2% HS-containing DMEM/F12 medium overnight. Cells were then treated with 10 ng/ml Activin A for 30 min, 1, 2, 4, or 6 h, respectively. Control cells were treated with equal volume of the Activin A solvent. To study the effects of small molecule inhibitors on Activin A-induced gene expression, MII cells were serum starved in 0.2% HS-containing DMEM/F12 overnight and then treated with 1 μM EW-7197 (TargetMol), 1 μM SB-505124 (TargetMol) or 10 ng/ml Activin A alone or in combination (as indicated in respective figures). Control cells were treated with equal amounts of DMSO, or Activin A solvent or both, accordingly. Whole cell lysates were extracted after 1 h of treatment and subjected to Western Blotting analysis. Similarly, to study the effects of small molecule inhibitors on IL13Rα2 expression, MIV cells were cultured for 24 h in the presence of 1 μM EW-7197 or SB-505124 and then total RNA was isolated, reverse transcribed into cDNA and subjected to quantitative real-time PCR (qPCR).

### Cloning of shRNA-Expressing Vector and Stable Transfection of MIV Breast Cancer Cells

To generate lentiviral vectors expressing small hairpin RNA (shRNAs) against INHBA and Smad2, AgeI/EcoRI-digested pLKO.1-puro vector was ligated with 58 base pair-oligos, as previously described (30), using the sequences listed in Supplementary Table 1. Establishment of MIV cells stably expressing shRNA constructs was performed by lentiviral-mediated transduction. Briefly, 293T cells were co-transfected with 5 μg pLKO-shScrambled (SCR), or pLKO-shINHBA or pLKO-shSmad2 with 3 μg psPAX2 and 1 μg pMD2.G plasmids. After 48 h, MIV cells were transduced with virus-containing medium in the presence of 10 μg/ml polybrene, selected with 2 μg/ml puromycin for 7–10 days and pooled for further assays.

### Protein Isolation and Western Blotting

For protein expression analysis, total cell lysates were obtained using radio immunoprecipitation assay (RIPA) buffer containing a protease inhibitor cocktail tablet (Roche), as well as the phosphatase inhibitors NaF (5 mM) and Na_3_VO_4_ (2 mM). Protein concentration was determined using the BCA protein assay kit (Pierce) and whole cell lysates were separated on a 10% polyacrylamide gel and transferred to a PVDF membrane. Membrane was blocked in 5% non-fat milk or BSA in TBS-T buffer and then incubated overnight at 4°C with anti-Inhibin βA (INHBA) (1:500, Santa Cruz Biotechnology), anti-IL13Rα2 (1:500, Santa Cruz Biotechnology), anti-phospho-Smad2 (Ser465/467, 1:1,000), anti-total Smad2 (1:1,000), anti-phospho-Smad3 (Ser423/425, 1:1,000), anti-total Smad3 (1:1,000), anti-phospho-Erk1/2 (1:1,000), anti-total Erk1/2 (Thr202/Tyr204, 1:1000), anti-phospho-Akt (Ser473, 1:1,000), anti-total Akt (1:1,000), anti-total STAT6 (1:1,000), anti-phospho-STAT6 (Tyr641, 1:1,000) (Cell Signaling Technology), β-actin (1:2,000), β-tubulin (1:2,000), or GAPDH (1:2,000) (Santa Cruz Biotechnology) antibodies followed by incubation with respective horseradish peroxidase (HRP)-conjugated secondary antibodies for 1 h at room temperature. The detection of secondary antibodies was performed with enhanced chemiluminescent system (Pierce) using ChemiDoc Gel Documentation system (BioRad). Protein expression was quantified using ImageJ software.

### RNA Isolation and Quantitative Real Time PCR

Total RNA was extracted from cancer cells using Trizol (Invitrogen) and reverse transcribed to cDNA using Superscript Reverse Transcriptase (Invitrogen). Quantification of gene expression was performed by real-time PCR using SYBR Fast Universal qPCR Master Mix (KAPA Biosystems) in a real-time PCR detection system (CFX96, BioRad). The β*-actin* housekeeping gene was used as internal control. Each biological sample was measured in triplicate for each gene. The relative quantification of gene expression was analyzed by the ΔΔCt quantification method, as previously described (30). The target gene sequences for real-time PCR primers are listed in Supplementary Table 2.

### Kaplan–Meier Plotter Analysis

Kaplan–Meier plotter (www.kmplot.com), an *in silico* online tool, was used to predict distant metastasis-free survival (DMFS) of patients with breast cancer of all subtypes based on expression of *IL13R*α*2* (probe 206172_at) or *INHBA* (probe 210511_s_at) or *SMTN* (probe 209427_at) or *VEGFA* (probe 210512_s_at) or *GDF15* (probe 221577_x_at) or mean expression of both *IL13R*α*2* and *INHBA* genes combined. Affymetrix gene expression data from multiple annotated breast cancer studies are combined into this database from which we queried for associations between expression of selected genes and patient outcomes (31).

### Scratch Wound Assay

MIV-shSCR and MIV-shINHBA breast cancer cells were cultured in complete medium and allowed to form a continuous monolayer. Cell-free space was then created by gently generating a wound using a 200 μl pipette tip. Cells were washed twice with Phosphate Buffered Saline (PBS) and allowed to migrate for 16 h. Images from at least four different fields were taken using an inverted microscope (Nikon TS100) at 0 and 16 h. Quantification of cell-free area (mm^2^) at different time points was performed using the Image J software and expressed as percentage (%) of wound closure.

### Transwell Migration Assay

Migration assays were performed by using 24-well transwell plates containing 8.0 μm pore transmembrane (Greiner BioOne). Serum-free DMEM/F-12 and 10% HS-containing DMEM/F-12 medium was added in the upper and bottom chamber, respectively, and 3 × 10^5^ MIV-shSCR cells or MIV-shINHBA cells were plated on the upper insert membrane. Cells were then allowed to migrate for 36 h, fixed with 4% paraformaldehyde, and stained with crystal violet (0.4%). Migrated cells localized on the bottom membrane surface were imaged and counted using an inverted microscope (Nikon TS100, 10 × magnification, at least four fields per condition).

### *In vitro* Cell Proliferation Assay

5 × 10^4^ MIV-shSCR and MIV–shINHBA cells were seeded in a 6-well plate and were subjected to a cell viability test using Alamar Blue reagent (Thermo), according to the manufacturer's instructions. Absorbance at 570/600 nm was measured at 24, 30, and 48 h post-cell seeding.

### *In vivo* Experiments

*In vivo* tumorigenesis assays were performed by orthotopically implanting 5 × 10^5^ MIV-shSCR or MIV-INHBA cells suspended in 40 μl phosphate buffer saline (PBS) in the fourth inguinal mammary fat pad of 6-week old female NOD.CB17-*Prkdc*^*scid*^/J mice (Harlan, UK). During the course of each experiment, tumor volume was measured twice a week using a digital caliper and calculated using the volume of an ellipsoid and assuming that the third dimension, *z*, is equal to $\sqrt{xy}$. Therefore, the volume was given by the equation: $V=\frac{4\pi }{3}\frac{\left(xyz\right)}{8}$. At the end of each experiment (35 days) mice were euthanized and tumors were excised, weighted and subjected to histological analysis. For metastasis assays, 6-week old female NOD.CB17-*Prkdc*^*scid*^/J mice were injected with 1 × 10^6^ MIV-shSCR or MIV-INHBA cells resuspended in 200 μl PBS via the lateral tail vein. Mice were monitored daily until they began to develop cachexia symptoms or weight loss. At the end of each experiment (~13 weeks) mice were euthanized, lungs were excised, macroscopically visible lung metastases were counted and subjected to histological analysis. *In vivo* tumorigenesis and metastasis assays were independently performed twice. All *in vivo* experiments were conducted in accordance with the animal welfare regulations and guidelines of the Republic of Cyprus and the European Union (European Directive 2010/63/EE and Cyprus Legislation for the protection and welfare of animals, Laws 1994-2013) under a license acquired and approved (No CY/EXP/PR.L1/2016) by the Cyprus Veterinary Services committee, the Cyprus national authority for monitoring animal research for all academic institutions.

### Histological and Immunofluorescence Analysis

Lungs excised from mice injected with either MIV-shSCR or MIV-INHBA breast cancer cells were fixed in 4% paraformaldehyde (PFA) and embedded in paraffin. Transverse tissue sections (8 μm-thick) were produced using an SRM200 microtome (Sakura), followed by staining with hematoxylin and eosin (H&E) using standard methodology. Bright-field images of stained slides were obtained using an Olympus BX53 microscope. For immunofluorescence analysis, tissue sections were re-hydrated and incubated in blocking solution (10% donkey serum, 3% FBS in PBS). Then sections were stained overnight with primary mouse anti-INHBA (1:50) and mouse anti-IL13Rα2 (1:50) antibodies (Santa Cruz Biotechnology) followed by incubation with secondary Alexa-fluor 488 donkey anti-mouse antibodies (1:300) and DAPI. Images were captured using an Olympus BX53 microscope. Average size of lung metastases was calculated from histology images using Image J software.

### Statistical Analysis

Results are represented as mean ± standard error (SE). Significant changes were determined by Student's *t*-test using two-tailed distribution. Differences with *p* < 0.05 were considered as significant (indicated by an asterisk ^*^).

## Results

### INHBA Is Overexpressed in Metastatic Breast Cancer Cells and Is Associated With Poor Metastasis-Free Survival

**Figure 1:**
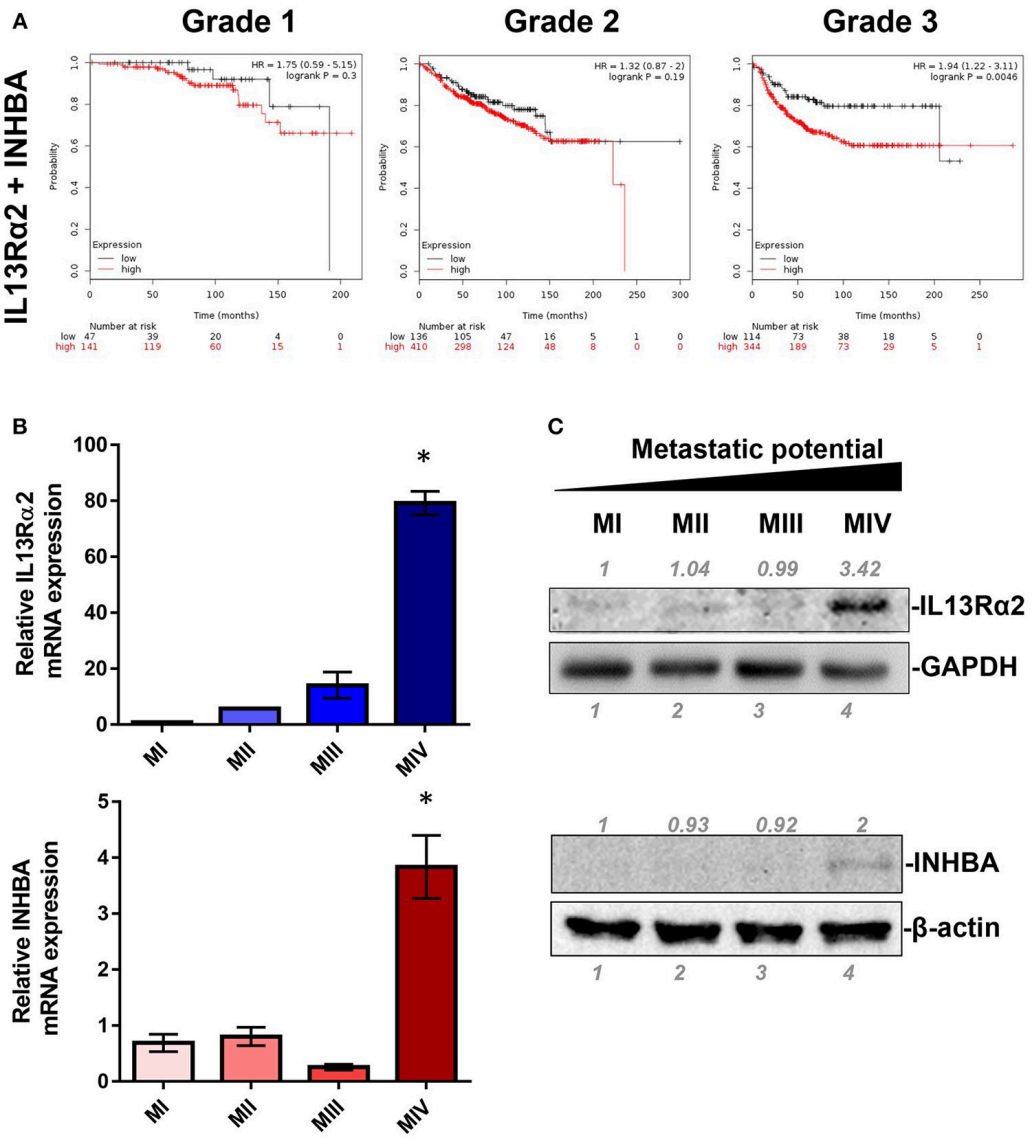
Combined high expression of INHBA and IL13Rα2 is correlated with poor prognosis of breast cancer. **(A)** Kaplan–Meier survival analysis for assessment of DMFS based on combined tumor *IL13R*α*2* and *INHBA* expression in 188 patients with Grade 1, 546 patients with Grade 2 and 458 patients with Grade 3 breast cancer. Survival curves were generated using the Kaplan–Meier Plotter online tool based on data stratified at the lower quartile (lowest 25% mean expression of both *IL13R*α*2* and *INHBA* combined vs. all others) (31). Curves were compared by log-rank test. **(B)** Real time qPCR was used to measure the mRNA expression of *IL13R*α*2* and *INHBA* in *four* breast cancer cell lines derived from common genetic background that exhibit distinct metastatic properties. The mean fold change was calculated and plotted for each gene. The expression in each sample was analyzed using the ΔΔCt method normalized against β-actin expression. Asterisk (^*^) indicates a statistically significant difference (*n* = 3; *p* < 0.05). **(C)** Western blotting analysis showing IL13Rα2 and INHBA protein levels in all cell lines. Antibodies against GAPDH and β-actin were used to verify equal protein loading, respectively. Protein expression was quantified using ImageJ software.

IL13Rα2 has recently emerged as an important driver for metastatic dissemination of breast cancer cells to the lungs and was also associated with reduced metastasis-free survival of breast cancer patients (14, 15, 32). In our previous studies, we exploited a well-established cell line model system for breast cancer progression which consists of the non-tumorigenic MCF10A (MI) mammary epithelial cell line and three of its derivatives, namely MII, MIII, and MIV cells obtained after serial passaging in nude mice (33), which exhibit distinct tumorigenic and metastatic properties *in vivo*; MII form benign hyperplasia, MIII form low-grade, well-differentiated carcinomas, whereas MIV develop high-grade, poorly differentiated carcinomas that metastasize to the lungs (34). Our published gene expression microarray analysis of these cell lines indicated that *IL13R*α*2* clusters together in a group of 29 genes including 4 genes encoding for secreted factors, *INHBA, SMTN, VEGFA*, and *GDF15*, which are overexpressed in the metastatic MIV vs. the non-metastatic cells (14, 29). Therefore, we initially hypothesized that one of these secreted proteins may control IL13Rα2 expression. To refine this list of candidate molecules we investigated the prognostic significance of these genes in primary breast tumors. Using an online tool, we performed meta-analysis of publicly available microarray datasets from breast cancer patients to generate Kaplan–Meier survival curves (31). Patients were separated into two groups based on the expression levels of *IL13R*α*2* or *INHBA* or *SMTN* or *VEGFA* or *GDF15* genes and the probability of DMFS over time was calculated. We found that higher expression of *IL13R*α*2* and *INHBA* could individually predict worse DMFS of breast cancer patients with grade 3 but not with grade 1 or grade 2 tumors, while this was not observed for *SMTN, VEGFA* or *GDF15* genes (Supplementary Figure S1). Since INHBA homodimer forms the secreted Activin A molecule (20), we hypothesized that this cytokine may act upstream to induce IL13Rα2 levels in an autocrine or paracrine fashion. To test this hypothesis, we investigated whether concomitant overexpression of INHBA and IL13Rα2 in breast cancer patients can be a significant prognostic factor by separating patients based on combined high or low expression levels of IL13Rα2 and INHBA in each patient. This analysis indicated that high mean expression of both genes in each patient with grade 3 tumors can predict worse DMFS with even stronger association (Figure 1A) than each gene alone (Supplementary Figure S1). Furthermore, we examined the correlation between INHBA and IL13Rα2 expression with the metastatic potential of breast cancer cells, using the established breast cancer cell line model system described above. By analyzing the gene and protein expression levels of all four cell lines, we validated that INHBA and IL13Rα2 mRNA and protein expression are significantly increased in the highly metastatic MIV cell line compared to its non-metastatic counterparts (Figures 1B,C), which was in agreement with our previous study (14). Collectively, these data supported the notion that there could be a potential functional correlation between *INHBA* and *IL13R*α*2* expression in patients.

### Activin A Promotes IL13Rα2 Expression in Breast Cancer Cells

**Figure 2:**
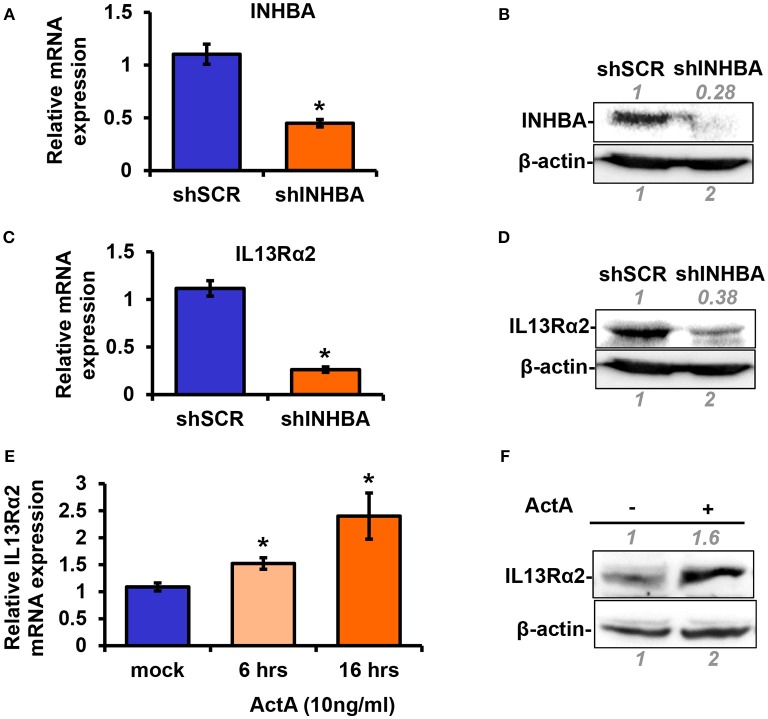
Activin A promotes IL13Rα2 expression in breast cancer cells. **(A,B)** Highly metastatic MIV cells were stably transduced with lentiviral vectors expressing shSCR or shINHBA. Real time qPCR and Western blotting analysis were used to compare mRNA and protein levels of INHBA, respectively. **(C,D)** Western blotting showing INHBA and IL13Rα2 protein levels in MIV-shSCR and MIV-shINHBA cells. Asterisk (^*^) indicates a statistically significant difference between MIV-shSCR and MIV-shINHBA cells (*n* = 3; *p* < 0.05). **(E)** Non-tumorigenic MII cells were cultured in medium containing 5% horse serum (HS) overnight and then treated with 10 ng/ml Activin A (ActA) for 6 and 16 h. Real time qPCR was used to measure the mRNA expression of IL13Rα2. Asterisk (^*^) indicates a statistically significant difference between Activin A-treated and mock-treated cells (*n* = 6; *p* < 0.05). **(F)** Western blotting showing protein expression of IL13Rα2 in MII cells treated with 10 ng/ml Activin A for 24 h. In all Western blots, antibody against β-actin was used to verify equal protein loading. Protein expression was quantified using ImageJ software.

Based on the data described above suggesting a plausible connection between *INHBA* and *IL13R*α*2* expression levels (Figure 1), we proceeded to directly address this question. To this end, using lentiviral-mediated shRNA transduction, we stably knocked down INHBA expression in the metastatic MIV cells to generate MIV-shINHBA as well as corresponding control cells (MIV-shSCR). Real-time PCR and Western blotting analysis indicated INHBA was effectively depleted at both mRNA and protein levels (Figures 2A,B). Importantly, this also resulted in a significant downregulation of IL13Rα2 mRNA and protein (Figures 2C,D), suggesting that INHBA could promote IL13Rα2 expression. To further validate this finding, we next treated non-metastatic MII cells with human recombinant Activin A followed by real-time PCR and Western blotting analysis. Our results showed that the expression of IL13Rα2 in MII cells is increased in response to Activin A treatment in a time-dependent manner (Figures 2E,F), suggesting that Activin A could induce IL13Rα2 expression in breast cancer cells.

### Smad Signaling Mediates Activin A-Induced IL13Rα2 Expression

**Figure 3:**
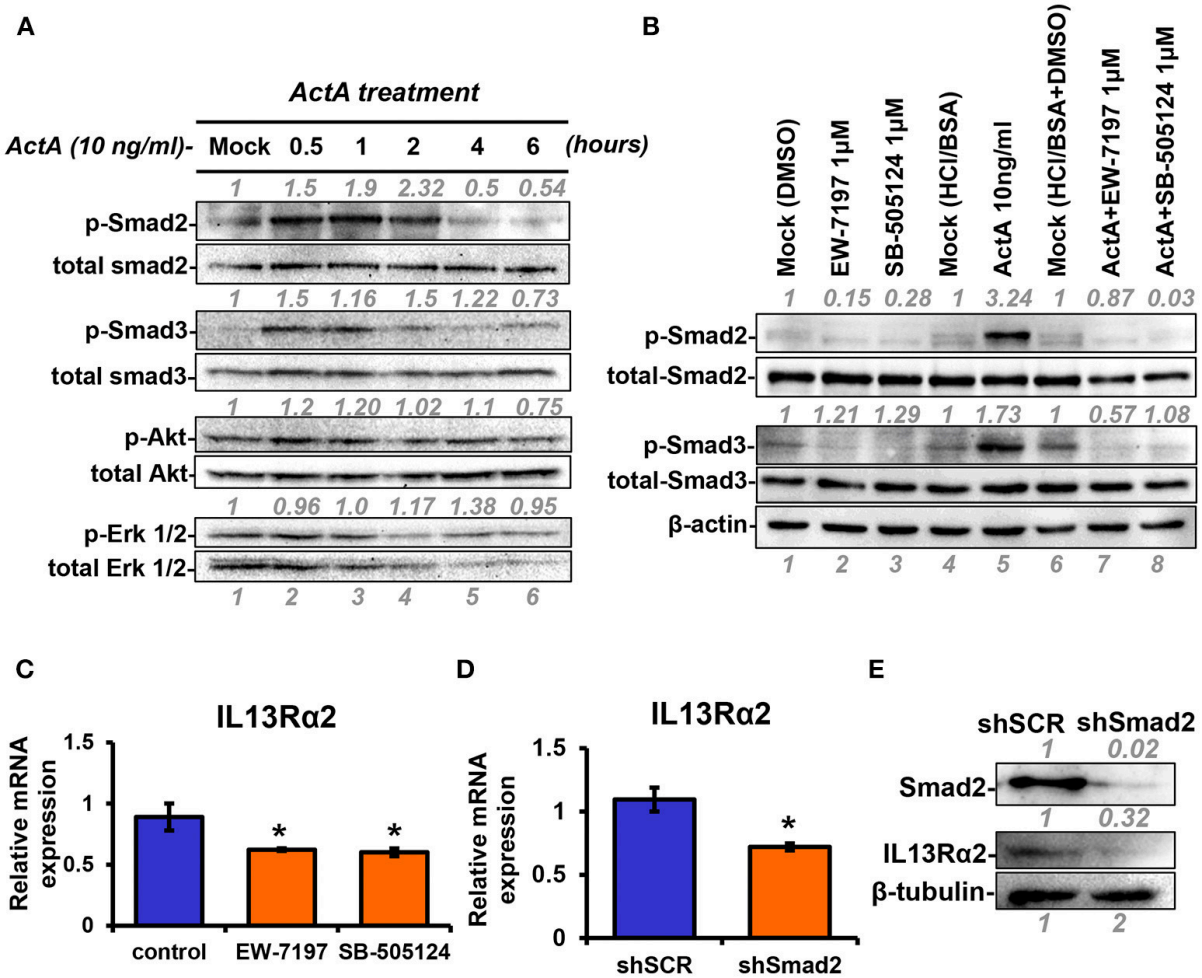
INHBA is correlated with IL13Rα2 expression in breast cancer cells. **(A)** MII cells were cultured in DMEMF/12 medium containing 0.2% horse serum (HS) overnight and then treated with 10 ng/ml Activin A for different time points. Western blotting was performed using whole cell lysates to assess phosphorylation of Smad2 (Ser465/467), Smad3 (Ser423/425), Akt (Ser 473), and Erk1/2 (Thr202/Tyr204). Detection of total Smad2, Smad3, Akt, and Erk1/2 was used as loading controls. Protein expression was quantified using ImageJ software. **(B)** MII cells were mock-treated (DMSO) or treated 1 μM EW-7197 or SB-505124, in the presence or absence of 10 ng/ml Activin A for 1 h. Western blotting was performed using whole cell lysates to assess the phosphorylation status of Smad2 and Smad3. Total Smad2, Smad3, and β-actin were detected as loading controls. **(C)** MIV cells were treated with 1 μM EW-7197 or 1μM SB-505124 for 24 h. Real time qPCR was used to measure the mRNA expression of IL13Rα2. Asterisk (^*^) indicates a statistically significant difference between Activin A-treated and mock-treated cells (*n* = 6; *p* < 0.05). **(D)** MIV cells were stably transduced with lentiviral vectors expressing shSCR or shSmad2. Real time qPCR was used to measure the mRNA expression of IL13Rα2. Asterisk (^*^) indicates a statistically significant difference between MIV-shSCR and MIV-shSmad2 (*n* = 3; *p* < 0.05). **(E)** Western blotting showing the protein expression of Smad2 and IL13Rα2 in MIV-shSCR and MIV-shSmad2 cells. Antibody against β-tubulin was used as a loading control. Protein expression was quantified using ImageJ software.

Next, to unravel the intracellular signaling pathways that mediate upregulation of IL13Rα2 by Activin A, we treated MII cells with Activin A and performed Western blotting analysis at different time points to examine the phosphorylation status of Smad2, Smad3, Akt, and Erk1/2 that were previously reported to be activated in response to this cytokine (28, 35). We found that Activin A could strongly induce phosphorylation of Smad2, in a time-dependent manner, which peaked at 2 h post-treatment. On the other hand, Smad3 and Akt were only modestly activated compared to mock-treated cells whereas Erk1/2 activation status remained unaffected (Figure 3A). As mentioned previously, Activin A functions through binding to the ActRII-ALK4 or ActRII-ALK2 receptor complex to initiate Smad-dependent or independent signaling cascades. To investigate whether Smad2 activation could also be regulated by increased ALK2 or ALK4 expression, we performed expression analysis upon Activin A treatment. We found that predominantly ALK2, and to a lesser extend ALK4, were increased 24h post-treatment, suggesting a potential role in sustaining Smad signaling activation (Supplementary Figures S2A,B). On the other hand, Activin A had no effect on IL13Rα1 or STAT6 phosphorylation (Supplementary Figures S2C,D). Therefore, we then used two different type I receptor (ALK4/ALK5) small molecule inhibitors, EW-7197 or SB-505124 to directly assess the contribution of Activin receptors in the activation of Smad signaling in breast cancer cells. Western blotting analysis revealed that both inhibitors could effectively abolish Activin-induced Smad2 and Smad3 phosphorylation, suggesting that ALK4 is necessary for their activation (Figure 3B). Since these two inhibitors could block Smad signaling, we investigated their effects on IL13Rα2 expression. We observed a significant downregulation of IL13Rα2 mRNA levels in the presence of each inhibitor, suggesting that Activin A/ALK4/Smad signaling axis is required for IL13Rα2 overexpression in metastatic MIV cells (Figure 3C). To confirm this finding, we also stably knocked down Smad2 in MIV cells, since this pathway exhibited the strongest activation among all intracellular mediators tested in response to Activin A. Interestingly, we found that IL13Rα2 expression was significantly suppressed upon Smad2 depletion at both mRNA and protein levels (Figures 3D,E). To assess whether Smad3 could also play a role in IL13Rα2 regulation we used SIS3, a specific small molecule inhibitor of Smad3 (36). Treatment of MIV cells confirmed that SIS3 could inhibit Activin A-induced Smad3 but not Smad2 phosphorylation (Supplementary Figure S3A). Interestingly, while treatment with the EW-7197 inhibitor could suppress IL13Rα2 mRNA levels, SIS3 had no effect (Supplementary Figure S3B). This evidence suggested that Activin A could act predominantly through a Smad2-dependent mechanism to regulate IL13Rα2 expression.

### Targeting INHBA Expression Impairs Migration, Delays Tumor Growth, and Suppresses Metastasis of Breast Cancer Cells to the Lungs

**Figure 4:**
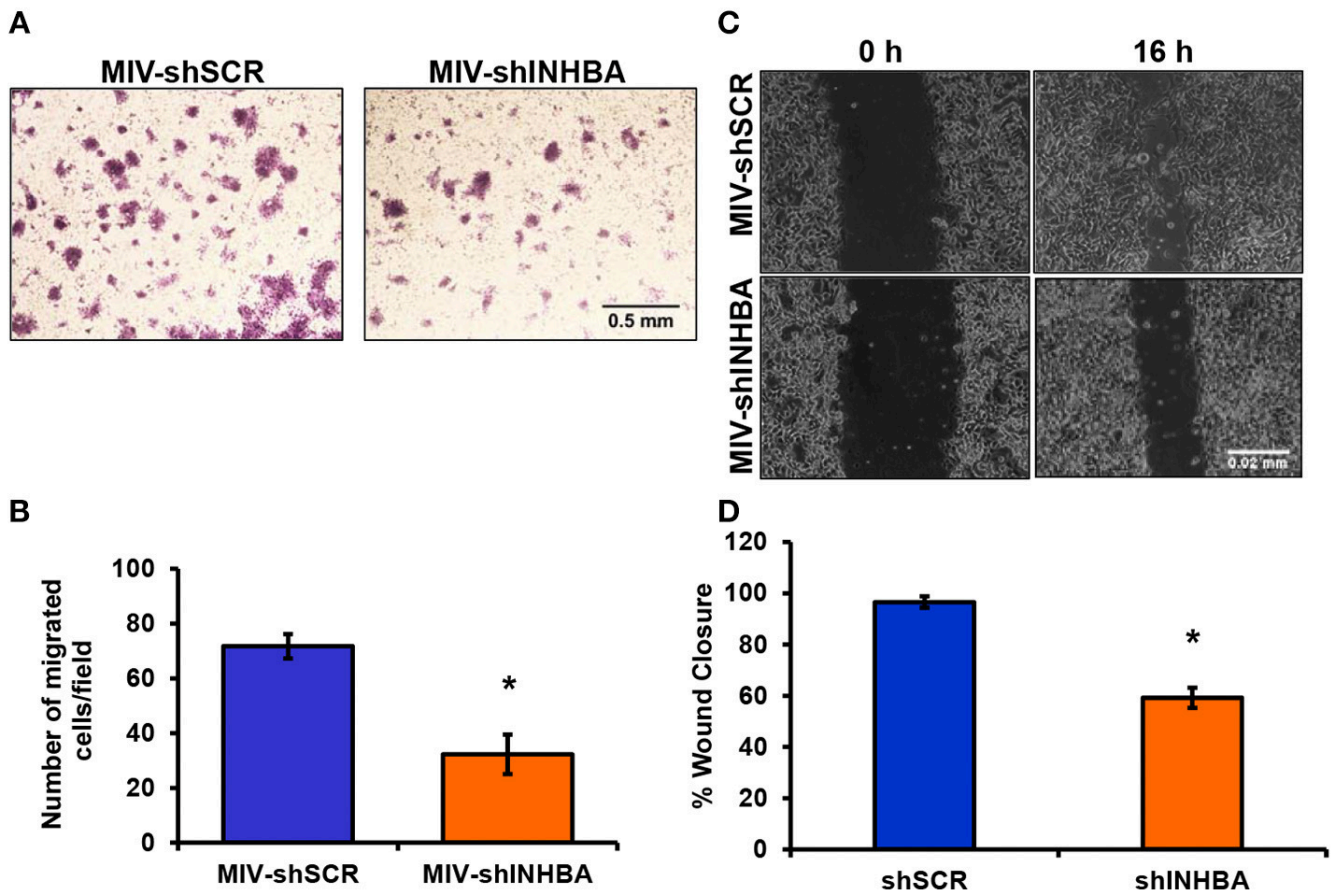
INHBA depletion impairs breast cancer cell migration. **(A)** Representative images from transwell migration assay used to quantify the migratory potential of MIV-shSCR and MIV-shINHBA cells for 36 h (4x magnification, scale bar 0.05 mm). **(B)** Graph showing the number of migrated MIV-shSCR per field compared to MIV-shINHBA breast cancer cells. Asterisk (^*^) indicates a statistically significant difference (*n* = 4; *p* < 0.05). **(C)** Representative images from scratch wound healing assay to assess the migratory ability of MIV-shSCR compared to MIV-shINHBA breast cancer cells for 16 h (10x magnification, scale bar 0.02 mm). **(D)** Graph showing the percentage (%) of wound closure of MIV-shSCR compared to MIV-shINHBA breast cancer cells, calculated as percentage (%) of cell-free area using Image J software. Asterisk (^*^) indicates a statistically significant difference (*n* = 3; *p* < 0.05).

**Figure 5:**
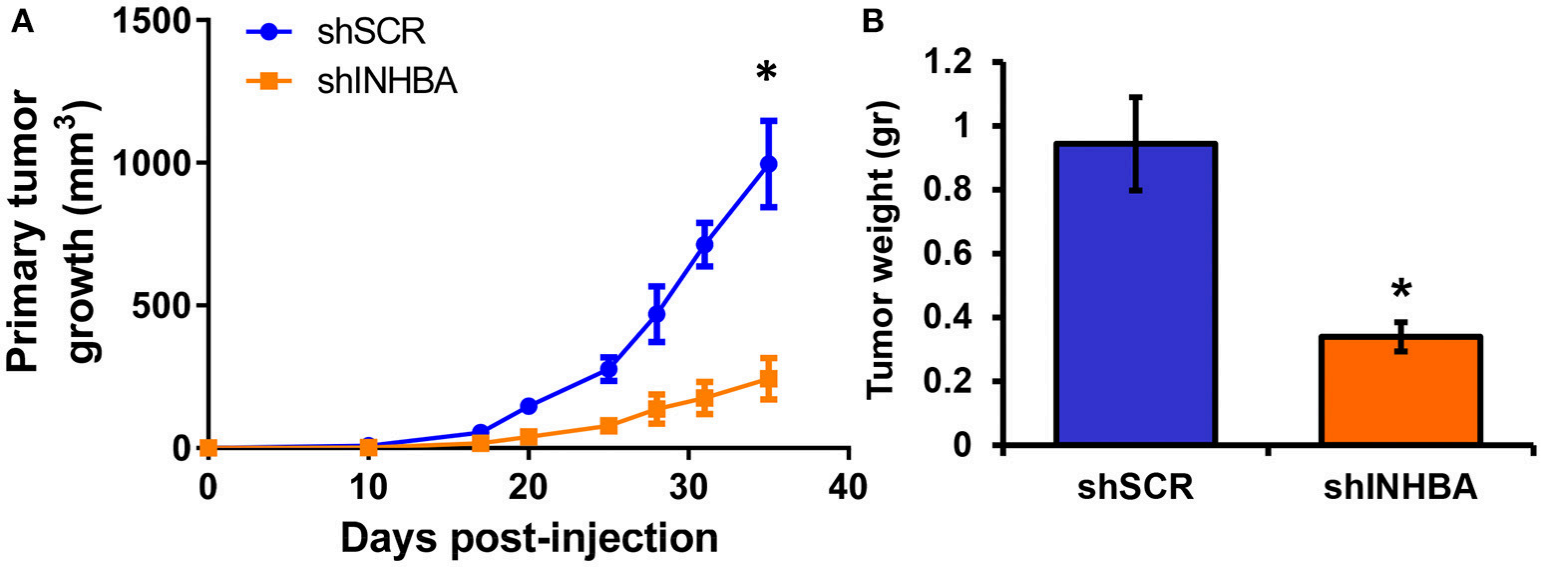
INHBA knockdown delays growth of primary breast tumors. **(A)** Comparison of growth rates of tumors derived from orthotopic implantation of MIV-shSCR or MIV-shINHBA breast cancer cells in NOD/SCID mice. Asterisk (^*^) indicates a statistically significant difference (*n* = 6; day 35; *p* < 0.05). **(B)** Tumor weight of primary MIV-shSCR and MIV-shINHBA breast tumors excised at the experimental endpoint. Asterisk (^*^) indicates a statistically significant difference (*n* = 6; day 35; *p* < 0.05).

**Figure 6:**
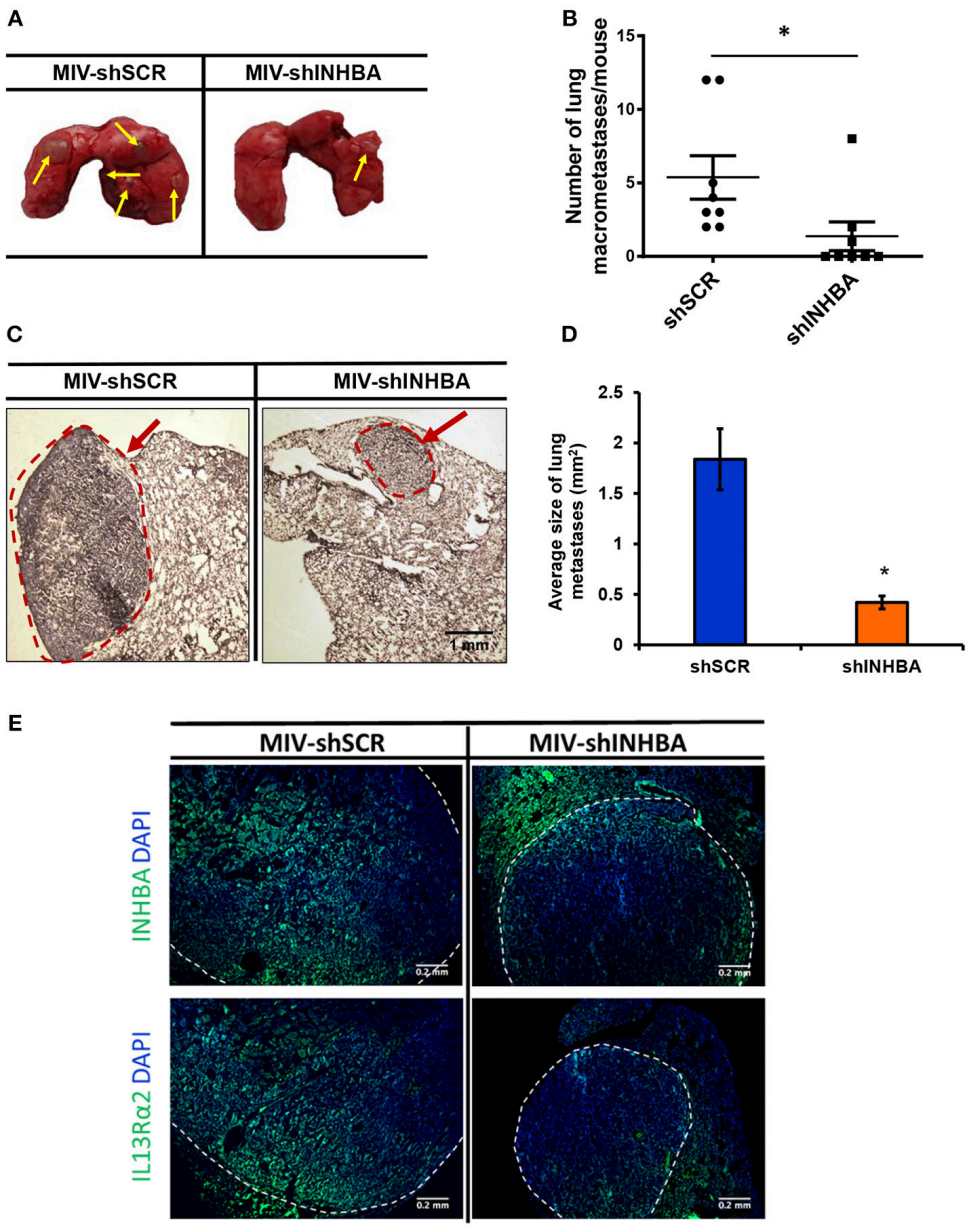
Targeting INHBA suppresses breast cancer metastasis to the lungs. **(A)** Representative images of lungs excised after euthanasia of mice injected intravenously with MIV-shSCR or MIV-shINHBA cells. Yellow arrows depict the presence of macrometastatic nodules. **(B)** Graph represents the number of macrometastases in the lungs of mice injected with MIV-shSCR and MIV-shINHBA cells. Asterisk (^*^) indicates a statistically significant difference (*n* = 8; week 13; *p* < 0.05). **(C)** Representative images from H&E staining of sections from paraffin-embedded lungs isolated from mice injected with MIV-shSCR or MIV-shINHBA cells. Red circle and arrow indicate the presence of metastatic lesions in lungs from MIV-shINHBA-injected mice (scale bar 1 mm, 4 × magnification). **(D)** Graph showing the average size of lung metastases (in mm^2^) formed by MIV-shSCR or MIV-shINHBA breast cancer cells, as calculated from histology images using the Image J software. Asterisk (^*^) indicates a statistically significant difference (*n* = 5). **(E)** Representative images from immunofluorescence staining of lung tissue sections indicating the expression levels of INHBA and IL13Rα2 in metastatic tumors formed by MIV-shSCR or MIV-shINHBA cancer cells. White lines depict the areas of metastatic tumors (scale bar 0.2 mm, 20 × magnification).

Since depletion of IL13Rα2 was previously shown to inhibit migration of metastatic breast cancer cells (14), we hypothesized that inhibition of Activin A signaling could have a similar effect on MIV cells. Interestingly, we found that MIV-shINHBA cells exhibited significantly lower migratory ability compared to MIV-shSCR cells, as indicated by both transwell migration (Figures 4A,B) and scratch wound healing assays (Figures 4C,D). To rule out potential proliferation-mediated effects in cell migration, MIV-shSCR and MIV-shINHBA cells were subjected in *in vitro* proliferation assay. Our findings indicated that depletion of INHBA had no effect in cell proliferation *in vitro* (Supplementary Figure S4), further supporting the notion that INHBA depletion impairs the migratory potential of metastatic breast cancer cells. Based on our previous studies suggesting that inhibition of IL13Rα2 delays tumor growth and suppresses formation of lung metastases (14), we wanted to investigate whether depletion of INHBA has a similar effect on metastatic MIV cells. To address this, we performed *in vivo* tumorigenesis assays by orthotopically implanting MIV-shSCR or MIV-shINHBA cells in the mammary fat pad of SCID mice. We found that upon INHBA knockdown, primary tumor growth was delayed compared to control cells (Figure 5A). Consistent with this finding, corresponding tumor weight was ~3-fold decreased when INHBA was depleted (Figure 5B). Most importantly, *in vivo* metastasis assays revealed that MIV-shINHBA cells exhibited significantly diminished metastatic potential compared to MIV-shSCR as demonstrated by the decrease in the number of macrometastases observed in the lungs of mice injected intravenously with MIV-shSCR or MIV-shINHBA cells (Figures 6A,B). Histological analysis using H&E staining and tumor area quantification further confirmed that not only the number but also the size of lung metastases was smaller when mice were injected with the MIV-shINHBA cells compared to controls (Figures 6C,D). Finally, immunofluorescence analysis on tissues from lung metastases confirmed that secondary tumors derived from INHBA-depleted cells exhibited lower levels of both INHBA and IL13Rα2 (Figure 6E), further supporting the notion that there is a functional link between these two proteins *in vivo*.

## Discussion

While metastatic dissemination of breast cancer cells to secondary organs is the main cause of patient fatality, the underlying molecular mechanisms remain far from fully elucidated. The identification of IL13Rα2 as a critical gene driving metastatic progression of the aggressive BLBC has generated hope for the development of more effective treatment approaches. However, the upstream molecular events that promote IL13Rα2 overexpression in a subset of BLBCs that could also potentially serve as alternative therapeutic targets are currently unknown. To address this, we took advantage of a well-established model system for breast cancer progression, comprising four cell lines with common genetic background but distinct tumorigenic and metastatic potential. By further analyzing our previously published gene expression microarray data from these cell lines (14, 29), we found that both *IL13R*α*2* and *INHBA* exhibit increased expression levels in metastatic compared to non-metastatic breast cancer cells and that overexpression of both genes can similarly predict worse DMFS of patients with grade 3 tumors. This is consistent with previous reports suggesting that INHBA overexpression or increased Activin A plasma levels are associated with poor prognosis in lung, gastric, pancreatic and esophageal cancers (37–40). Our data presented here also demonstrate that shRNA-mediated depletion of INHBA in metastatic breast cancer cells led to suppression of IL13Rα2 mRNA and protein levels, whereas treatment of non-metastatic cells with Activin A resulted in upregulation of IL13Rα2 expression. Moreover, while Activin A could strongly induce Smad2 phosphorylation, knocking down Smad2 or treatment with ALK4/ALK5 small molecule inhibitors abolished downstream signaling activation and downregulated IL13Rα2 levels. This is consistent with numerous studies suggesting that Activin A predominantly mediates intracellular signaling *via* binding to ALK2/ALK4 receptors and Smad phosphorylation (18, 41). Furthermore, we show here that knocking down INHBA in metastatic breast cancer cells, decreased their migratory potential without affecting their proliferative capacity *in vitro*, delayed primary tumor growth formation and significantly suppressed formation of lung metastases *in vivo*. The differential effect on cancer cell proliferation *in vitro* and *in vivo* suggests that the inhibitory effect of INHBA knockdown in tumor growth *in vivo* may involve, to some extent, interactions with the tumor microenvironment. Moreover, our data are supported by a recent study demonstrating that exogenous overexpression of Activin A promotes epithelial-mesenchymal transition, invasion, tumor growth, and metastasis of breast cancer (28). Our findings are also in agreement with evidence showing that reduced expression of the Activin A inhibitor Follistatin (FSH) was associated with worse survival of patients with HER2-positive breast cancers and enhanced formation of lung metastases *in vivo* (42). Moreover, the ALK4/ALK5 small molecule inhibitor EW-7197 has been reported to potently suppress breast cancer metastasis to the lungs (43), suggesting that its effects could be mediated, at least in part, through downregulation of IL13Rα2 expression.

**Figure 7:**
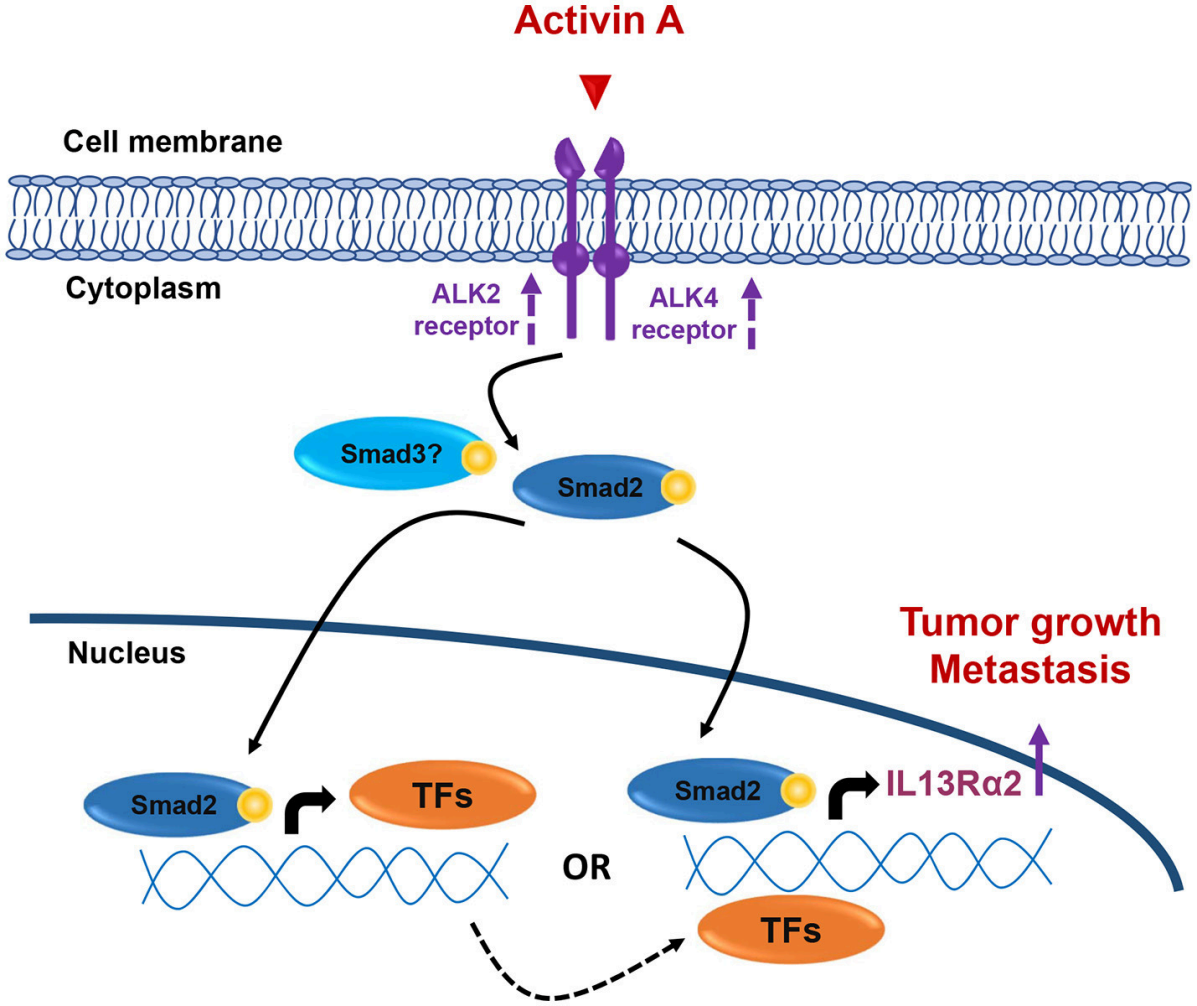
Working hypothesis model. INHBA overexpression in metastatic BLBCs results in increased synthesis and secretion of Activin A ligand that may act both locally and/or systemically. This results in activation of Smad2 signaling that could induce IL13Rα2 expression either indirectly via upregulation of intermediate transcription factors (TFs) which, in turn, can increase IL13Rα2 levels or directly via binding of phosphorylated Smad2 on IL13Rα2 promoter. Increase in ALK2 and ALK4 receptor levels may also be implicated in sustaining long-term activation of Smad signaling. Overexpression of IL13Rα2 protein levels could then elicit tumorigenic, pro-migratory and pro-metastatic effects on breast cancer cells resulting in formation of lung metastases, as previously described (14).

Collectively, our data presented here suggest that overactive Activin A signaling may promote tumorigenesis, migration and lung metastasis of breast cancer, at least in part, by inducing directly or indirectly IL13Rα2 expression in a Smad2-depended manner (Figure 7), without excluding the possibility that additional downstream effector genes could also be involved in this process. Therefore, the development of therapeutic strategies using small molecule inhibitors against Activin receptors or neutralizing antibodies targeting Activin A ligand, could be used as an alternative approach for breast tumors overexpressing INHBA and/or IL13Rα2.

## Author Contributions

MK performed *in vitro* experiments, interpreted the data, wrote and critically revised the manuscript. FM performed *in vivo* experiments, wrote and critically revised the manuscript. CW performed *in vitro* experiments, wrote and critically revised the manuscript. MP performed histological analysis, wrote and critically revised the manuscript. SO performed *in vitro* experiments, wrote and critically revised the manuscript. ST directed the project, read and critically revised the manuscript and TS directed the project, read and critically revised the manuscript. PP conceived the hypothesis, directed the project, performed *in vivo* experiments, interpreted the data, wrote and critically revised the manuscript. All authors read and approved the final manuscript.

### Conflict of Interest Statement

The authors declare that the research was conducted in the absence of any commercial or financial relationships that could be construed as a potential conflict of interest.
