# Supplementary material for: Activin A Signaling Regulates IL13Rα2 Expression to Promote Breast Cancer Metastasis
Source: Front Oncol. 2019 Feb 5;9:32. doi: 10.3389/fonc.2019.00032 (PMC6370707; doi:10.3389/fonc.2019.00032)
Supplement: Supplementary file 1 [file Table_1.DOCX]

| **Supplementary Table 1. Sequences of shRNA oligos** | |
| --- | --- |
| **Gene name** | **Oligo sequence** |
| ***INHBA*** | Forward: 5’--3’ CCGGGGAGATAGAGGATGACATTGGCTCGAGCCAATGTCATCCTCTATCTCCTTTTTG  Reverse: 5’--3’ AATTCAAAAAGGAGATAGAGGATGACATTGGCTCGAGCCAATGTCATCCTCTATCTCC |
| ***Smad2*** | Forward: 5’--3’ CCGGGCCTGATCTTCACAGTCATCACTCGAGTGATGACTGTGAAGATCAGGCTTTTTG  Reverse: 5’--3’ AATTCAAAAAGCCTGATCTTCACAGTCATCACTCGAGTGATGACTGTGAAGATCAGGC |

| **Supplementary Table 2. Primers used for qPCR** | |
| --- | --- |
| **Primer Name** | **Primer sequence** |
| ***β-actin*** | Forward: 5’-CGAGCACAGAGCCTCGCCTTTGCC-3’  Reverse: 5’-TGTCGACGACGAGCGCGGCGATAT-3’ |
| ***INHBA*** | Forward: 5’-TTTCTGTTGGCAAGTTGCTG-3’  Reverse: 5’-CGGGTCTCTTCTTCAAGTGC-3’ |
| ***IL13Ra2*** | Forward: 5’-TCTTGGAAACCTGGCATAGG-3’  Reverse: 5’-TCTGATGCCTCCAAATAGGG-3’ |
| ***IL13Ra1*** | Forward: 5’- gtccctggtgttcttcctga-3’  Reverse: 5’- agtgtggaattgcgcttctt-3’ |
| ***ALK2*** | Forward: 5’-gcggtaatgaggaccactgt-3’  Reverse: 5’-ccctgctcataaacctggaa-3’ |
| ***ALK4*** | Forward: 5’-ggtgttcctcctgttcctca-3’  Reverse: 5’-ggagcgtcttgtctttggag-3’ |

**Supplementary Figure Legends:**

**Supplementary Figure S1: Increased INHBA and IL13Rα2 expression levels are correlated with poor prognosis of breast cancer.** Kaplan-Meier survival analysis for assessment of DMFS based on tumor *IL13RΑ2* or *INHBA* or *SMTN* or *VEGFA* or *GDF15* expression in 188 patients with Grade 1, 546 patients with Grade 2 and 458 patients with Grade 3 breast cancer. Survival curves were generated using the Kaplan-Meier Plotter online tool based on data stratified at the lower quartile (lowest 25% expression of each gene versus all others) (1). Curves were compared by log-rank test.

**Supplementary Figure S2: Activin A induces ALK2 and ALK4 expression.** MII cells were cultured medium containing 5% horse serum (HS) overnight and then treated with 10 ng/ml Activin A (ActA) for 6, 16 or 24hours. Real time qPCR was used to measure the mRNA expression of **(A)** ALK2, **(B)** ALK4 or **(C)** IL13Rα1. The expression in each sample was analyzed using the ΔΔCt method normalized against β-actin expression. Asterisk (*) indicates a statistically significant difference between Activin A-treated (24h) and mock-treated cells (n=3; p<0.05). **D.** Western blotting showing protein expression of phosphorylated STAT6 (Y641) in MII cells treated with 10 ng/ml Activin A for 24 hours. Blotting against total STAT6 was used to verify equal protein loading. Protein expression was quantified using ImageJ software.

**Supplementary Figure S3: Suppression of Activin A-induced Smad3 phosphorylation using SIS3 inhibitor does not affect IL13Rα2 expression. A.** MIV cells were cultured in medium containing 0.2% horse serum and were first pre-treated for 1 hour either with DMSO (mock) or with 1μΜ EW-7197 or with 1μΜ SIS3 or both, followed by treatment with 10 ng/ml Activin A or its solvent for 1 hour. Western blotting was performed using whole cell lysates to assess the phosphorylation status of Smad2 and Smad3. Total Smad2 and Smad3 were detected as loading controls. **B.** MIV cells were cultured in complete medium and treated either with DMSO (mock) or with 1μΜ EW-7197 or with 1μΜ SIS3 for 48 hours. Real time qPCR was used to measure the mRNA expression of IL13Rα2. The expression in each sample was analyzed using the ΔΔCt method normalized against β-actin expression. Asterisk (*) indicates a statistically significant difference between compared groups (n=3; p<0.05). Protein expression was quantified using ImageJ software.

**Supplementary Figure S4: INHBA depletion does not affect *in vitro* cell proliferation.** *In vitro* cell viability assay using Alamar Blue was performed to assess the proliferation rates of MIV-shSCR or MIV-shINHBA cells. Absorbance at 570/600nm was measured at 24h, 30h and 48h post-cell seeding.

**References:**

1. B. Gyorffy, A. Lanczky, A. C. Eklund, C. Denkert, J. Budczies, Q. Li and Z. Szallasi: An online survival analysis tool to rapidly assess the effect of 22,277 genes on breast cancer prognosis using microarray data of 1,809 patients. *Breast Cancer Res Treat*, 123(3), 725-31 (2010) doi:10.1007/s10549-009-0674-9
