# Supplementary figures and images for: Activin A Signaling Regulates IL13Rα2 Expression to Promote Breast Cancer Metastasis
Source: Front Oncol. 2019 Feb 5;9:32. doi: 10.3389/fonc.2019.00032 (PMC6370707; doi:10.3389/fonc.2019.00032)

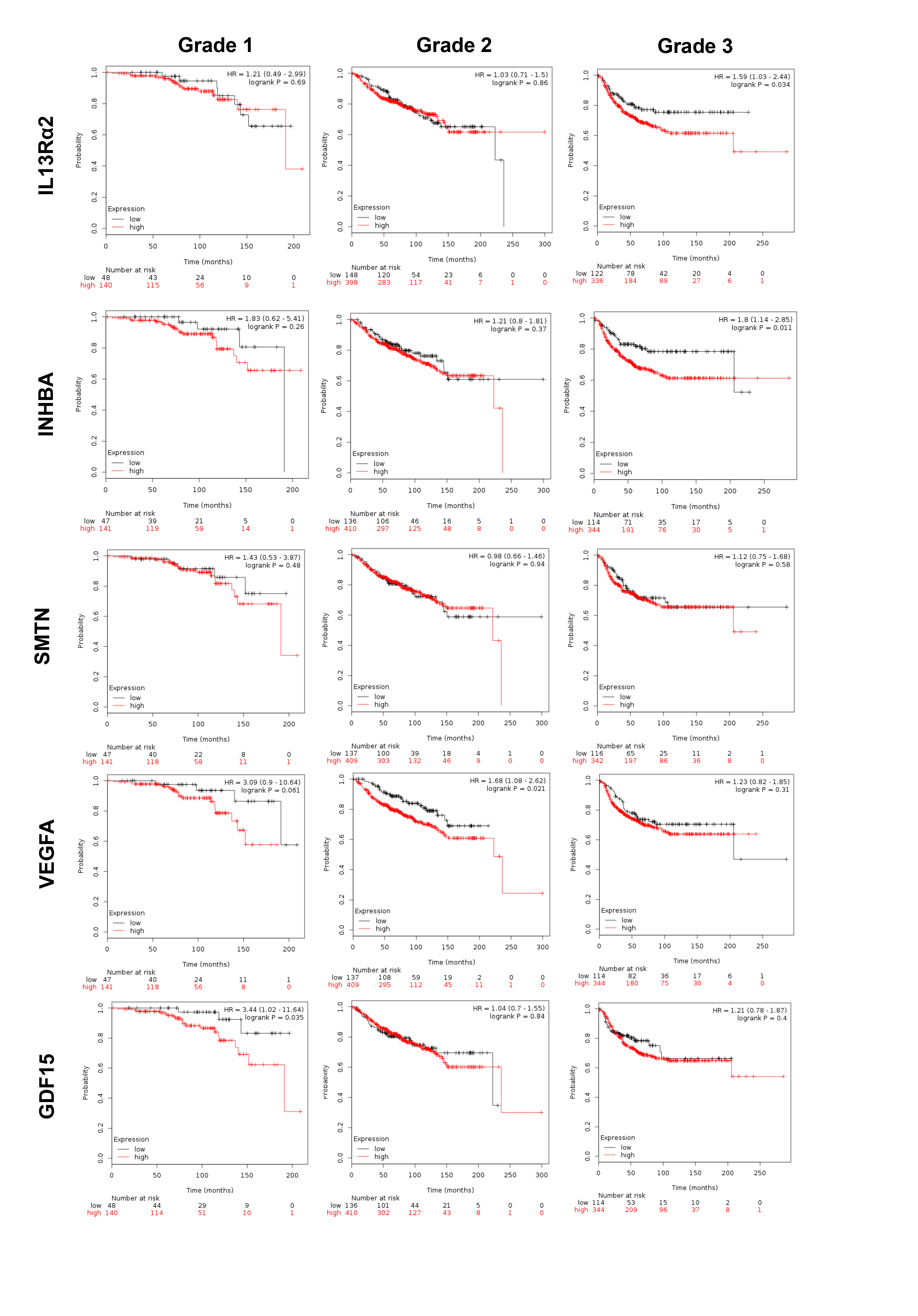

Supplement: Supplementary file 2 [file Image_1.TIF]

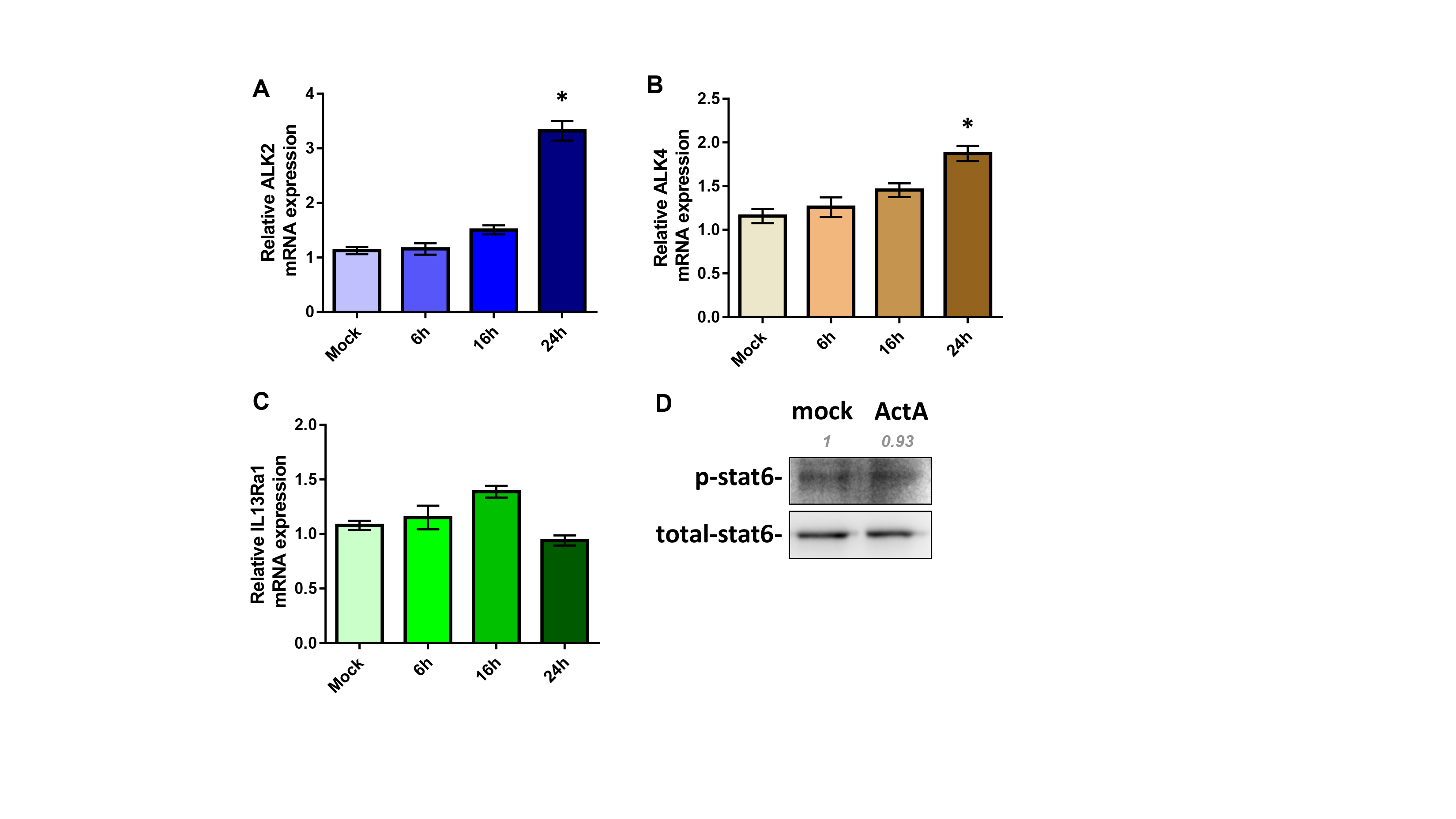

Supplement: Supplementary file 3 [file Image_2.TIF]

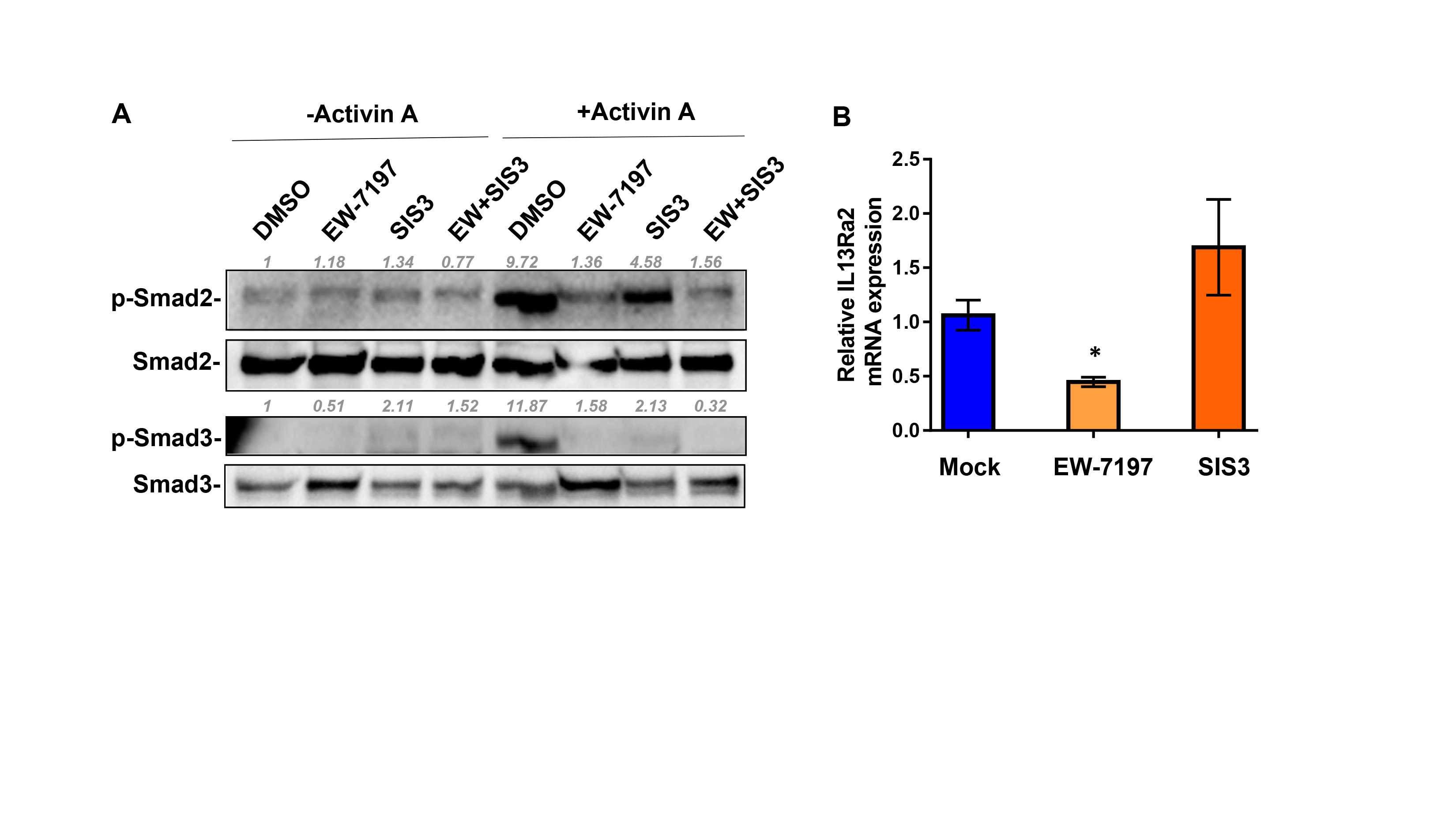

Supplement: Supplementary file 4 [file Image_3.TIF]

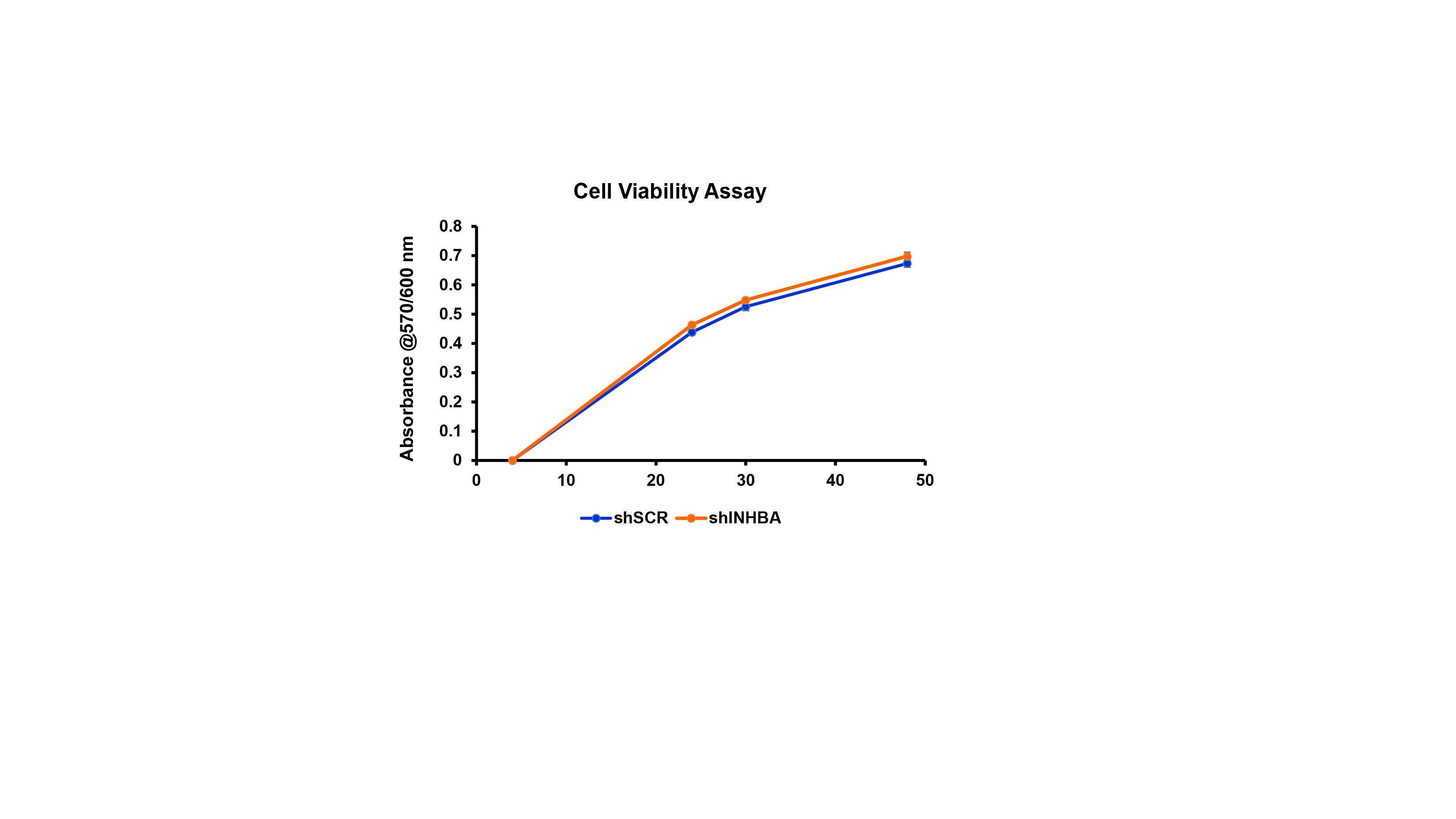

Supplement: Supplementary file 5 [file Image_4.TIF]
